# Visual Evoked Potentials in Rett Syndrome

**DOI:** 10.15844/pedneurbriefs-29-10-7

**Published:** 2015-10

**Authors:** J. Gordon Millichap

**Affiliations:** 1 Division of Neurology, Ann & Robert H. Lurie Children's Hospital of Chicago, Chicago, IL; 2 Departments of Pediatrics and Neurology, Northwestern University Feinberg School of Medicine, Chicago, IL

**Keywords:** Rett Syndrome, MECP2 Gene, VEP, Cortical Processing Deficits

## Abstract

Investigators from the Boston Children's Hospital recorded pattern-reversal visual evoked potentials (VEPs) in Mecp2 heterozygous female mice and in 34 girls with Rett syndrome (RTT).

Investigators from the Boston Children's Hospital recorded pattern-reversal visual evoked potentials (VEPs) in Mecp2 heterozygous female mice and in 34 girls with Rett syndrome (RTT). The amplitudes and latencies of VEP waveform components were quantified, and were related to disease stage, clinical severity, and MECP2 mutation type in RTT patients. Visual acuity was also assessed in mice and patients by modulating the spatial frequency of the stimuli.

Mecp2 heterozygous female mice and RTT patients exhibited a similar decrease in VEP amplitude, most striking in the later stages of the disorder. RTT patients showed a slower recovery from the principal peak of the VEP response that was impacted by MECP2 mutation type. Both patients and mice displayed a deficit in discriminating small patterns when the spatial frequency of the stimulus was increased, indicating a lower visual spatial acuity in RTT.

In conclusion, VEP may be used to assess brain function across species and in children with severe disabilities like RTT. The findings support the introduction of standardized VEP analysis in clinical and research settings to probe the mechanism underlying functional impairment and to monitor progression of the disorder and response to treatment. [1]

COMMENTARY. VEP analysis using a checkerboard pattern stimulus appears to provide a promising method of evaluation of cortical functioning in patients with Rett syndrome. Factors that may weaken the significance of VEP analysis as a biomarker of cortical functioning in RTT include the occurrence of seizures and rarely, pattern-sensitive epilepsy. About 60% of RTT have epilepsy, usually with onset between 3 and 20 years of age [2], and 5% of children with epilepsy in general may have pattern sensitive seizures [3, 4]. Epilepsy, diagnosed in 45% of patients in the above study, was treated with antiepileptic drugs that may have modified VEP responses, but this risk was considered to be small [1]. Studies of auditory brainstem responses in Rett syndrome at the Kennedy Krieger Institute, Baltimore, conclude that sedation can cause prolongation of the I-V interpeak latency intervals, and cautious interpretation of evoked potentials is warranted if sedated control groups are not used for comparison [5]. Reports of altered auditory and somatosensory processing suggest that the present VEP impairments represent a global cortical deficit in RTT [1].

Finally, the authors cannot explain a contribution of any ophthalmic abnormalities to the cortical deficits reported. A study of visual function in RTT at Glasgow Caledonian University, UK, found substantial refractive errors were common and all 11 subjects with RTT had pattern-onset VEPs. Latencies and amplitudes did not differ from those in 18 normal controls [6]. In future studies, a complete eye exam will be incorporated in the study design at the time of the VEP recording [1].
